# Incidence of Gastrointestinal Bleeding After Percutaneous Coronary Intervention: A Single Center Experience

**DOI:** 10.14740/cr322w

**Published:** 2014-02-27

**Authors:** Fahad Aziz

**Affiliations:** Penn State Hershey Medical Center, 500 University Drive, MC, Hershey, PA 17033, USA. Email: fahadaziz.md@gmail.com

**Keywords:** Incidence, Gastrointestinal bleeding, Percutaneous coronary intervention

## Abstract

**Background:**

Gastrointestinal (GI) bleeding is a hemorrhagic complication after percutaneous coronary intervention in patients with acute myocardial infarction. The purpose of the study is to determine predictors of GI bleeding and impact of GI bleeding on the patients undergoing percutaneous coronary intervention.

**Methods:**

GI bleeding occurred in 6 (7.1%) of 84 patients with STEMI/NSETMI (ST-segment elevated myocardial infarction/Non ST-segment elevated myocardial infarction) undergoing primary percutaneous coronary intervention.

**Results:**

Univariate analysis demonstrates that patients with GI bleeding had a significantly higher previous GI bleeding (16.66% vs. 8.6%, P < 0.001). Higher Killip classification at presentation was associated with higher incidence of GI bleeding (61% vs. 18%, P < 0.01). The use of proton pump inhibitors did not reduce the risk of GI bleeding. The GI bleeding in these patients was associated with higher mortality and morbidity in the post percutaneous coronary intervention period.

**Conclusion:**

Although, GI bleeding in patients with MI significantly increases mortality and morbidity, previous GI bleeding and higher Killip class are associated with higher incidence of GI bleeding. High-risk patients for GI bleeding can be identified at presentation.

## Introduction

The use of combination of antiplatelets (Aspirin and clopidogrel) and anticoagulants is well established in patients with ST-segment elevated myocardial infarction (STEMI) and Non ST-segment elevated myocardial infarction (NSTEMI). According to ACC/AHA guidelines, the patients with STEMI and NSTEMI should received a full dose of aspirin and should be loaded with clopidogrel on presentation and should receive anticoagulation with unfractionated or low molecular weight heparin for at least 48 hours [1].

The cardiac benefit from this therapy has also been paralleled by an increase in the incidence of bleeding complications, specially, gastrointestinal (GI) complications. Several authors have discussed the gastrointestinal bleeding in setting of STEMI and NSTEMI [2-8].

The purpose of this study is to determine the predictors of GI bleeding and its impact on outcomes in the patients presenting with STEMI and NSTEMI, undergoing percutaneous coronary intervention (PCI). The identification of these patients who are at increased risk of bleeding after PCI is urgently required so that strategies to reduce bleeding and thereby improve outcomes can be developed.

## Methods

This was a retrospective study. From August 2012 to December 2012, 84 patients underwent PCI for STEMI or NSTEMI.

STEMI was diagnosed when a patient had ST elevation in two consecutive leads of more than > 1 mm on electrocardiogram (EKG). NSTEMI was diagnosed with elevation of troponins with or with out ischemic changes on EKG other than ST elevations in patients presenting with chest pain. For evaluation of the peak serum levels of cardiac biomarkers, blood samples were obtained every 6 h for 48 h or until the level returned to normal.

Baseline renal insufficiency is defined as estimated glomerular filtration rate less than 60 mL/min per 1.73 m^2^, with estimated glomerular filtration rate calculated by using the Modification of Diet in Renal Disease Study equation developed in 1999 [9].

Killip classification was based on information regarding severity of heart failure and systolic blood pressure at admission. Specifically, Killip class I patients had no evidence of heart failure; Killip class II patients had mild heart failure with crackles involving one-third or less of the lung fields and systolic blood pressure of 90 mmHg or higher; Killip class III patients had pulmonary edema with crackles involving more than one-third of the lung fields and systolic blood pressure of 90 mmHg or more; and Killip class IV patients had cardiogenic shock with any crackles and systolic blood pressure of less than 90 mmHg.

All the patients underwent coronary angiography to confirm the diagnosis and for therapeutic intervention.

We compared baseline clinical features, medical therapy, and outcomes between study patients with and without GI bleeding and assessed independent correlates of GI bleeding.

GI bleeding was defined as the occurrence of GI bleeding during the start of initial antiplatelet and anticoagulant therapy, or within 1 week of discontinuation of anticoagulant therapy.

Significant GI bleeding was defined as clinically evident bleeding, including hematemesis, heme-positive coffee ground emesis, heme-positive melena, or occult causing a decrease in hemoglobin level of 1 g/dL or more without an identifiable extra-intestinal source.

A percutaneous transfemoral or tans-radial approach was used in all patients. Patients without prior antiplatelet therapy were pretreated with oral aspirin 335 mg, clopidogrel 300 mg or 600 mg, and intravenous heparin at the beginning of the presentation, and an additional bolus of heparin was administered to maintain activated clotting time greater than 300 seconds. If PCI was needed, intravenous heparin infusion was continued after PCI and was adjusted to maintain the activated partial thromboplastin time at 60 to 85 seconds for 24 - 48 h. Administration of platelet glycoprotein IIb/IIIa receptor inhibitor was left to the clinician’s discretion. After the procedure, all patients were treated with oral aspirin 81 mg and clopidogrel 75 mg daily for at least 1 month.

## Results

### Incidence of GI bleeding

From August 2012 to December 2012, 84 patients (55 males and 29 females) with a mean age of 63.2 years underwent PCI for acute MI (5 STEMI and 79 NSTEMI). Six patients developed GI bleeding during the combination of anti-platelet and anticoagulation therapy, yielding an incidence of 7.1%.

### Predictive factors for GI bleeding

**Figure 1:**
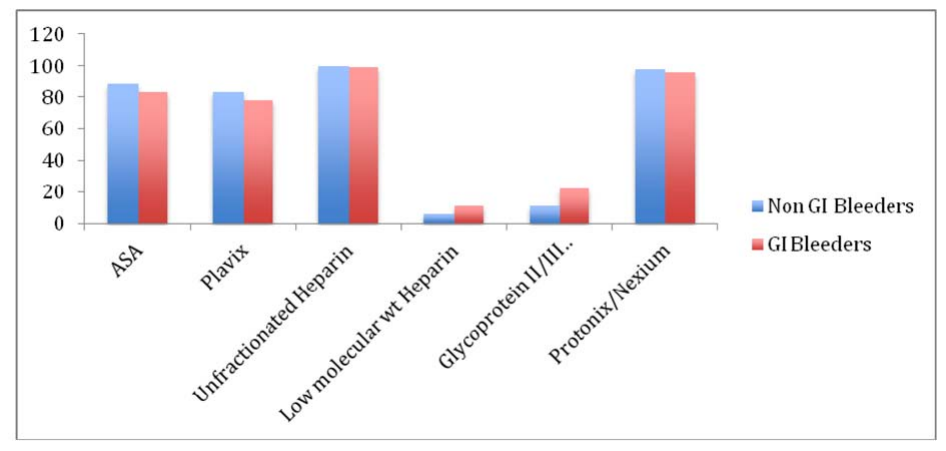
Comparison of medications in 2 groups.

The differences in the clinical characteristics and medication in the patients with or without GI bleeding were not significant (Fig. 1).

The two groups did not differ significantly in age, sex, or history of underlying disease such as hypertension, diabetes mellitus, and heart failure.

**Figure 2:**
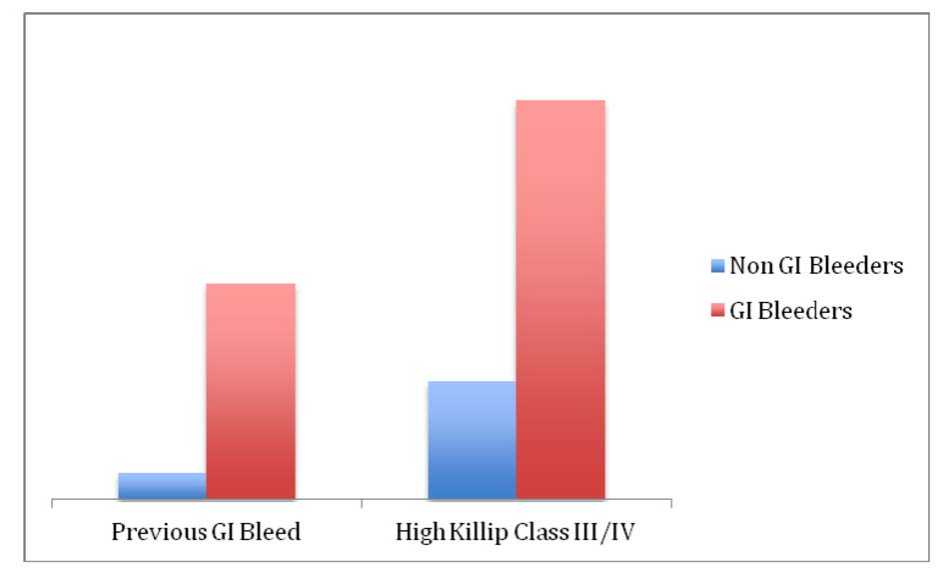
Previous history of GI bleed and higher killip class are associated with high risk of GI bleed.

Univariate analysis demonstrates that patients with GI bleeding had a significantly higher previous GI bleeding (16.66% vs. 8.6%, P < 0.001). Higher Killip classification was associated with higher incidence of GI bleed (61% vs. 18%, P < 0.01) (Fig. 2).

The prophylactic prescription of proton pump inhibitors (PPIs) did not appear to be protective against GI bleeding in the present study (22% vs. 13%, P = 0.22) (Fig. 2).

To analyze the significance of these variables further and examine if a predictive relationship in patients with GI bleeding and MI exists, multivariate analysis was completed. Multivariate analysis indicated that previous GI bleeding (odds ratio, 22.1; 95% confidence interval, 5.61 - 86.89; P < 0.001) was significant independent predictive factor for occult GI bleeding in patients with MI (Fig. 2).

### Outcome

GI bleeding was associated with significantly prolonged stays in the intensive care unit (mean (SD), 5.4 (6.7) days vs. 3.6 (3.6) days, P = 0.04); but similar total hospital stays (8.8 (8.6) days vs. 7.7 (5.6) days, P = 0.43). The in-hospital mortality of patients with GI bleeding was higher than the in-hospital mortality in patients without GI bleeding (44% and 9%, P < 0.001).

## Discussion

Our analysis of the cohort of the patients presenting with STMI and NSTEMI showed the incidence of GI bleeding of 7.1% in the post PCI period. Previous studies have shown that 0.7% to 3.0% of patients with acute coronary syndrome had GI bleeding [2-4]. Patients in our study appeared to have a higher incidence of GI bleeding. Our study also showed that the previous history of GI bleeding and higher Killip classification at presentation were strongly associated with the incidence of heart failure. On the same time the use of PPI’s did not reduce the risk of GI bleeding. The incidence of GI bleeding in these patients with STEMI/NSTEMI was associated with much greater mortality and morbidity.

### Clinical implication of the study

The present study has shown that GI bleeding in patients after PCI results in prolonged stays in the intensive care unit and mortality. Previous history of GI bleeding and higher Killip classification at presentation might be predictive of GI bleeding in patients undergoing PCI. By identification of these high-risk patients, more aggressive measures should be taken to prevent GI bleeding in this group. It may also help in the identification GI bleed earlier enough to prevent significant mortality and morbidity [10].

This study had several limitations including being a retrospective study, it may have inherent shortcomings. None of the patient in our study underwent upper GI endoscopy, thus the exact site of bleeding remained unknown. The data of the PPI use in these patents was also inconsistent because some cardiologists did not feel comfortable in starting PPI’s in the patient with the recent PCI, so some patients didn’t receive PPI. Further the dose of PPI was not standardized.

### Conclusion

GI bleeding in the patients after PCI is associated with significantly higher mortality and morbidity. Previous GI bleeding and higher Killip at presentation were significant independent risk factors for GI bleeding. The use of PPI did not reduce the risk of GI bleeding. Further, studies will be needed to determine the most appropriate antiplatelet and anticoagulant therapy in the patients who are at increase risk of GI bleeding in post PCI period.
